# Hydroxylated Tetramethoxyflavone Affects Intestinal Cell Permeability and Inhibits Cytochrome P450 Enzymes

**DOI:** 10.3390/molecules29020322

**Published:** 2024-01-09

**Authors:** Kuo-Ching Jan, Mohsen Gavahian

**Affiliations:** Department of Food Science, National Pingtung University of Science and Technology, No. 1, Xuefu Rd, Neipu, Pingtung 91201, Taiwan; jankuoching@mail.npust.edu.tw

**Keywords:** polymethoxyflavones, tetramethoxyflavone, cytochrome P450, healthy food, flavonoids, bioactive compounds

## Abstract

Tetramethoxyflavones (TMFs) found in the *Citrus* genus have garnered considerable interest from food scientists and the health food industry because of their promising biological properties. Nonetheless, there are currently limited data available regarding the effectiveness and bioavailability of “hydroxylated TMFs”, which are flavones known for their potential in disease prevention through dietary means. This study aims to provide insights into the chemical and biological properties of hydroxylated TMF and evaluates its effects on intestinal cell permeability and cytochrome P450 (CYP) inhibition. Liquid chromatography–mass spectrometry (LC-MS) and microsomes analyze the TMFs and hydroxylated TMFs, elucidating cell penetration and metabolic inhibition potential. 3H7-TMF shows the fastest (1-h) transport efficiency in intestinal cells. The Caco-2 cell model exhibits significant transport and absorption efficiency. Dissolved hydroxyl-TMF with hydrophilicity possibly permeates the gut. 3H7-TMF has higher transport efficiency (46%) 3H6-TMF (39%). IC_50_ values of TMFs (78-TMF, 57-TMF, 3H7-TMF, 3H6-TMF) against CYP enzymes (CYP1A2, CYP2D6, CYP2C9, CYP2C19, CYP3A4) range from 0.15 to 108 μM, indicating potent inhibition. Hydroxyl groups enhance TMF hydrophilicity and membrane permeability. TMFs display varied inhibitory effects due to hydroxyl and methoxy hindrance. This study underscores the strong CYP inhibitory capabilities in these TMFs, implying potential food–drug interactions if used in medicines or supplements. These findings can also help with food nutrition improvement and pharma food developments through innovative approaches for *Citrus* waste valorization.

## 1. Introduction

*Citrus* genus (e.g., *Citrus sinensis*, *Citrus mangshanesis*, and *Citrus clementina*) holds significant importance as a fruit crop cultivated worldwide. However, the processing of citrus fruits results in the generation of substantial waste comprising peel, seeds, and pomace. This citrus waste contains highly bioactive substances and phytochemicals, such as essential oils, ascorbic acid, sugars, carotenoids, flavonoids, dietary fiber, polyphenols, and trace elements. These invaluable compounds serve as building blocks for the development of functional foods. Tetramethoxyflavones are a group of flavonoid compounds found in various plant sources, including certain foods. These compounds are known for their potential health benefits due to their antioxidant and anti-inflammatory properties. Citrus fruits such as oranges, grapefruits, and mandarins are known to contain tetramethoxyflavones. These compounds contribute to the physiological activity of these fruits [1,2,3]. In addition, Citrus fruits are known for their abundant content of polymethoxylated flavones (PMFs), such as sinensetin, tangeretin, nobiletin, 3,5,6,7,3′,4′-hexamethoxyflavone, 5,6,7,4′-tetramethoxyflavone (TMF), and 3,5,6,7,8,3′,4′-heptamethoxyflavone, from the genus *Citrus* which have a broad spectrum of biological features [4]. They contain multiple methoxyls with low polarities, flat structures, and potent biological activity [5]. PMFs have highlighted molecular mechanisms and signaling pathways for anti-inflammatory, anti-cancer, chemopreventive, antidiabetic, anti-obesity, hepatoprotective, and neuroprotective effects in neurodegenerative diseases [6]. Specifically, 5,7,3′,4′-TMF is chondroprotective by reducing endoplasmic reticulum stress (ERS)-induced chondrocyte death [7]. TMFs have been shown to have therapeutic effects relating to arthritis, diabetes mellitus, cancer, and neurodegenerative disorders, as well as liver, kidney, and heart diseases [8], in reducing the concentration of inflammatory cytokines TNF, IL-1β, and PGE_2_ in the knee synovial fluid, protecting cartilage via the downregulation of EP/cAMP/PKA signaling, and improving cholesterol dysregulation through increasing the expression of FOXO3a/LXRα/ABCA1 signaling via SIRT1 [7,9,10]. TMFs are also linked to the function of the ER sensor IRE1 and suppression of the EP/cAMP/PKA, ROS-AKT/mTOR, and β-catenin signaling pathways in osteoarthritic chondrocytes [9,11,12].

According to a prior study, TMFs’ vasorelaxant activity is linked to NO/sGC/cGMP and prostacyclin pathways, calcium and potassium channels, and muscarinic and beta-adrenergic receptors [13]. Nobiletin induces apoptosis and cell cycle arrest in cancer cells. It can suppress the migration and invasion of cancer cells by inhibiting epithelial-to-mesenchymal transition (EMT) and EMT-related factors such as TGF-β, ZEB, Slug, and Snail. In addition, NOB inhibits oncogene factors such as STAT3, NF-κB, Akt, PI3K, and Wnt [14]. Various in vitro and in vivo studies have documented the chemopreventive effects of hydroxylated PMFs, including anticancer, anti-inflammatory, anti-atherosclerosis, and neuroprotective properties. They regulate cell death, proliferation, differentiation, repair, and metabolism by modulating signaling cascades, gene transcription, protein functions, and enzyme activities. The mechanisms of action of hydroxylated PMFs in disease chemoprevention depend on their structure and the number and position of hydroxyl groups [15,16,17]. The 3′,4′,5,7-tetramethoxyflavone could be considered a relatively rare bioactive compound, but it is present in several plant materials with health benefits and disease prevention (Supplementary Materials Table S1). However, further research is needed to determine the efficacy of PMFs and hydroxylated PMFs in chemoprevention and oral bioavailability.

Biotransformation and pharmacokinetic reactions occur in the liver, intestine, kidneys, and lungs. The liver is the primary site of drug metabolism, with the gut performing a significant role. Cytochrome P450 (CYP) is one of the most critical enzymes required for the phase I biotransformation process, and its superfamily includes CYP1A2, CYP2C9, CYP2C19, CYP2D6, CYP2E1, and CYP3A4 [18]. They oxidize nonpolar molecules, increasing their polarity, and allowing them to pass through the urinary stream. These enzymes are responsible for the phase I oxidative biotransformation reaction. CYP3A4 is mainly involved in the metabolism of drugs, especially for medications administered via various routes. Genetic variations within CYP genes significantly influence drug efficacy. Among the major variants, genetic polymorphisms in the CYP2D6, CYP2B6, CYP1B1, and CYP2A6 genes have been highlighted [19]. Given the broad substrate specificity of the CYP from the herbal active constituents, there is an increased likelihood of possible interactions: the P-glycoprotein (P-gp)-related mechanism for flavonoid poor absorption. The presence of 3-OH, 5-OH, 3′-OCH3, and 4′-OCH3 groups is crucial for binding flavonoids to P-gp, as evidenced by the PMF structure–affinity binding. Flavonoid drugs often lead to an increase in P-gp expression, resulting in faster efflux and reduced absorption. These findings offer valuable insights into strategies for enhancing flavonol absorption [20]. Furthermore, the cytotoxicity of PMFs containing a hydroxyl group was significantly higher than PMFs lacking a hydroxyl group when tested against HeLa, A549, HepG2, and HCT116 cancer cell lines. The number and location of hydroxy and methoxy groups in PMF pharmacokinetics and metabolites were critical factors in their modulation [21]. The bioavailability of a compound is influenced by several factors, such as its solubility, stability, and potential interactions with food or other drugs. Hence, investigating the solubility and permeability of PMFs can provide important insights into their bioavailability and the relationship between their chemical structure and absorption. Natural substances that block CYP enzymes may have significant absorbed and metabolic impacts.

**Figure 1:**
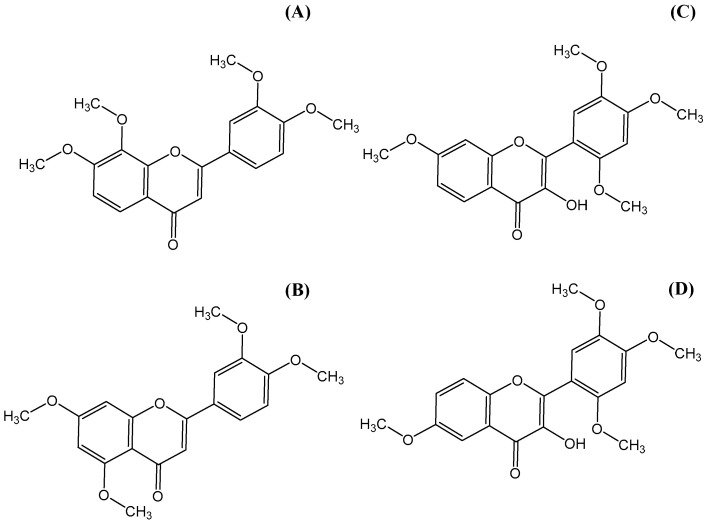
Chemical structures of (**A**) 3′,4′,7,8-tetramethoxyflavone (78-TMF), (**B**) 3′,4′,5,7-tetramethoxyflavone (57-TMF), (**C**) 3-hydroxy-2′,4′,5′,7-tetramethoxyflavone (3H7-TMF), and (**D**) 3-hydroxy-2′,4′,5′,6-tetramethoxyflavone (3H6-TMF).

To date, no definitive oral bioavailability or P450 inhibition of TMFs have been postulated, and limited data on gastrointestinal permeabilization and CYP inhibition have been reported. This study aimed to investigate the gastrointestinal permeabilization and inhibition of P450 isozymes by four different TMFs, presented in Figure 1, i.e., 3′,4′,7,8-TMF (78-TMF), 3′,4′,5,7-TMF (57-TMF), 3-hydroxy-2′,4′,5′,7-TMF (3H7-TMF), and 3-hydroxy-2′,4′,5′,6-TMF (3H6-TMF).

## 2. Results and Discussions

### 2.1. Analysis of TMFs in the Caco-2 Cell by LC-MS

**Figure 2:**
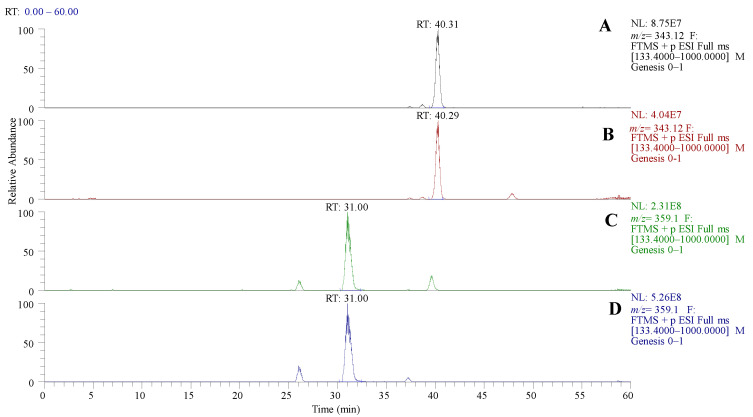
LCMS chromatogram of TMF. (**A**) 3′,4′,7,8-tetramethoxyflavone (78-TMF), (**B**) 3′,4′,5,7-tetramethoxyflavone (57-TMF), (**C**) 3-hydroxy-2′,4′,5′,7-tetramethoxyflavone (3H7-TMF), and (**D**) 3-hydroxy-2′,4′,5′,6-tetramethoxyflavone (3H6-TMF).

**Figure 3:**
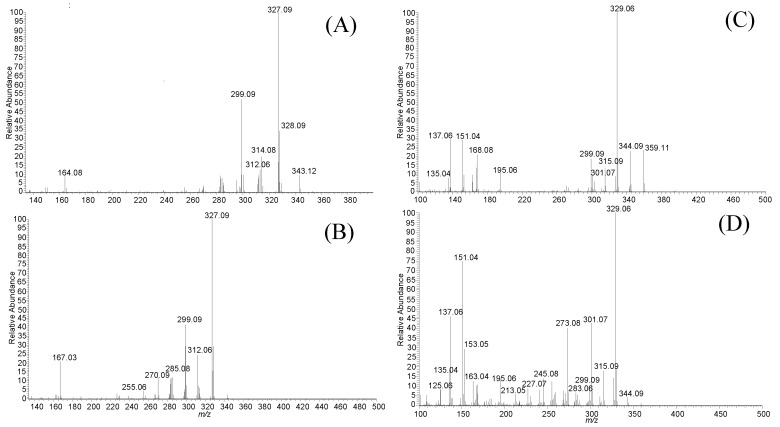
Mass fragmentation spectrum of TMF. (**A**) 78-TMF, (**B**) 57-TMF, (**C**) 3H7-TMF, and (**D**) 3H6-TMF.

A method for quantifying TMFs (78-TMF, 57-TMF, 3H7-TMF, and 3H6-TMF) by LC-MS was developed in this study. After conducting transport experiments using a Caco-2 cell monolayer model, this approach was used to examine TMFs on the apical, intracellular, and basolateral sides. Sample analyses of the retention time of each TMF [78-TMF (40.31 min), 57-TMF (40.29 min), 3H7-TMF (31.00 min), and 3H6-TMF (31.00 min)] was performed with UPLC (Figure 2). Specific m/Q values were selected, and the corresponding fragments were scanned to identify precursor ions of each TMF (78-TMF (164.08), 57-TMF (167.03), 3H7-TMF (299.09, 168.08), and 3H6-TMF (301.07, 273.08)) (Table 1 and Figure 3). This analytical approach was consistently applied in subsequent trials.

### 2.2. Transport of TMFs across Caco-2 Cell Monolayer

Caco-2 cell monolayers, known for resembling enterocyte-like human cancer cells, were used to test the absorption and transport mechanisms across the intestinal epithelium of functional foods and drugs. These cells can express morphological and functional characteristics of the small intestine, such as brush borders, tight junctions, intestinal efflux, and uptake transporters, which affect the passage of drugs and food extracts from the intestinal lumen to the bloodstream [22]. The transport studies using Caco-2 cell monolayers have revealed essential information about intestinal permeability, the transport method (paracellular, transcellular, or active carrier), the function of intestinal metabolism, and the impact of the P-glycoprotein, breast cancer resistance, and multidrug drug-resistance protein efflux system [23]. The minimum solubility required for an orally active drug is also determined by its intestinal permeability.

#### 2.2.1. The Transport Efficiency of TMFs

The transport experiments indicated that most TMFs remained on the apical side after 4 h, with minimal crossing to the basolateral side. The apical side showed more significant peak areas of TMFs compared to the basolateral side. Concentrations of 78-TMF, 57-TMF, 3H7-TMF, and 3H6-TMF on the basolateral side increased with time in the time-course investigation, indicating time- and size-dependent transportation. Notably, the concentration of 3H7-TMF observed on the basolateral side was higher than that of other TMFs at each time point, with distribution percentages of 37.58 ± 0.11, 34.00 ± 0.01, and 39.43 ± 2.50 for 78-TMF, 57-TMF, and 3H6-TMF at four hours, respectively. The efficiency of 3H7-TMF transport was significantly greater than other TMFs (*p* < 0.05) (Table 2). The second-highest distribution was observed in 3H6-TMF. The presence of a hydroxyl group facilitated the transport of TMFs across the Caco-2 cell monolayer, particularly during the early stages of the time-course research. Both 3H7-TMF and 3H6-TMF groups showed higher transfer efficiencies than 78-TMF and 57-TMF. At 1 and 4 h, the rise percentages were 18.54 and 22.40%, respectively.

#### 2.2.2. The Apparent Permeability Coefficient of TMFs

The Parallel Artificial Membrane Permeation Assay (PAMPA) gauges were used to assess a compound’s passive permeability [24,25]. The cumulative impact of transportation carried out by both absorptive and secretory transporters is represented by the apparent permeability (*P*_app_) from the apical (A) side to the basolateral (B) side. A chemical is considered to have excellent permeability when *P*_app_ > 1.0 × 10^−7^ cm/s. According to the literature, compounds with very low Caco-2 cell permeability (*P*_app_ less than 3 × 10^6^ cm/s) exhibited poor oral absorption, whereas those with high *P*_app_ values showed excellent oral absorption [26,27]. The permeability studies also revealed that hydroxyl-PMFs (3H6-TMF and 3H7-TMF) were transported more efficiently through the Caco-2 cell monolayer. The permeability values for 78-TMF, 57-TMF, 3H7-TMF, and 3H6-TMF were 8.83 × 10^5^ cm/s, 1.43 × 10^5^ cm/s, 1.95 × 10^6^ cm/s, and 1.74 × 10^6^ cm/s, respectively, showing that 3H7-TMF and 3H6-TMF were more effectively transported among the TMFs. The TEER values were also analyzed after completing the transport experiment, and they remained unaltered, showing that the additional TMFs had no deleterious effect on the Caco-2 cell monolayer. These findings are consistent with previous outcomes. However, the *P*_app_ of 57-TMF was only 1.43 × 10^5^ cm/s in the current permeability study, showing that it is poorly absorbed. In a study conducted on three PMFs, 3′-hydroxy-5,6,7,4′-tetramethoxyflavone, 3,5,6,7,8,3′,4′-heptamethoxyflavone, and 3-hydroxy-5,6,7,8,3′,4′-hexamethoxyflavone, all these PMFs exhibited excellent permeability and were not affected by efflux [28]. Our results show that TMFs generally have low solubility but are extremely permeable to membranes. The position of the hydroxyl group on the C-ring and the presence of methoxy groups on the A-ring significantly influence the solubility of PMFs [29]. PMFs have a medium to high overall permeability, indicating they may readily pass the phospholipid membrane. Their hydrophobic nature can be attributed to multiple methoxy groups, as opposed to typical flavonoids, which have several hydrophilic hydroxyl groups. Therefore, the total absorption of TMFs must consider both solubility and permeability. In the Caco-2 cell monolayer model, 3H7-TMF demonstrated greater transport and absorption efficiency than the other TMFs at each time point. The presence of methoxy at position 7C on ring A and hydroxy on ring C appears to improve the efficiency of permeability and solubility of 3H7-TMF, making it particularly beneficial for transport and absorption.

### 2.3. CYP Inhibitory Effect of TMFs In Vitro

#### 2.3.1. IC_50_ of CYP Affected by TMFs

**Figure 4:**
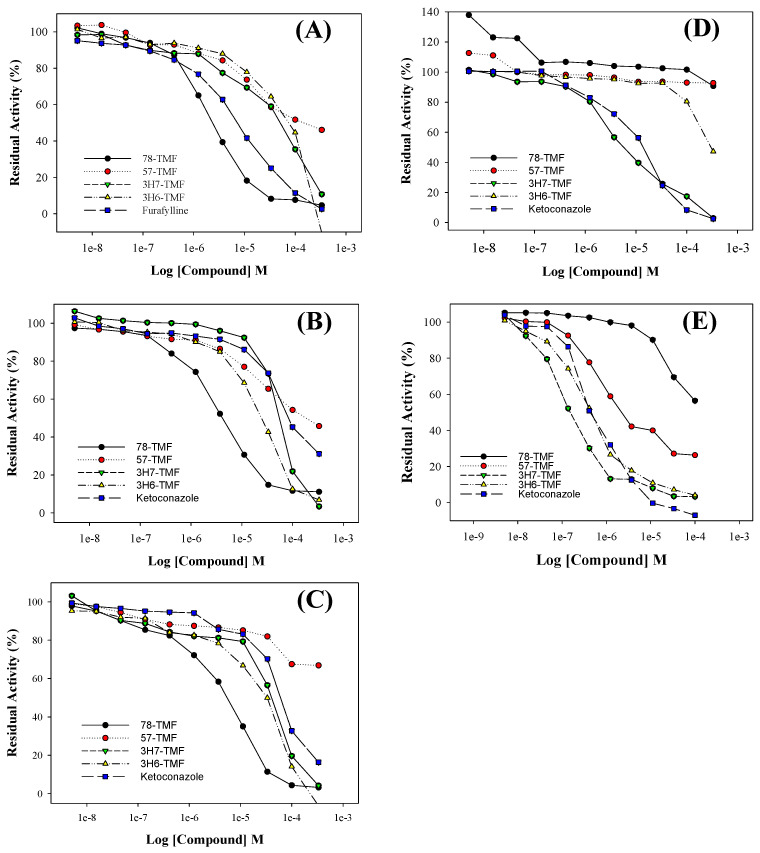
Dependence of inhibition activities catalyzed by CYP1A2 (**A**), 2C9 (**B**), 2C19 (**C**), 2D6 (**D**), and 3A4 (**E**) on the concentrations of TMF.

Metabolism primarily relies on the CYP450 enzyme and its isoforms, namely CYP 3A4, 2D6, 1A2, 2C9, and 2C19. These enzymes are responsible for detoxifying drugs as they pass through the liver [30]. CYP450 enzymes catalyze hydroxylation and demethylation processes on the C6-C3-C6 flavone skeleton, making CYP450 a major enzyme system involved in flavone metabolism. In human P450s, various flavonoids have been found to inhibit CYP1A1, CYP1A2, CYP1B1, CYP2C9, and CYP3A4 differently, depending on their number and position of hydroxyl and/or methoxy groups [31]. In this study, the inhibitory effects of TMFs (78-TMF, 57-TMF, 3H7-TMF, and 3H6-TMF) (Figure 4) were assessed by measuring their inhibitory impact on CYP 1A2, 2C9, 2C19, 2D6, and 3A4. The positive controls, ketoconazole and furafylline, had IC_50_ values of 0.13–3.54 μM. The inhibitory effects of 78-TMF, 57-TMF, 3H7-TMF, and 3H6-TMF on CYP 1A2, 2C9, 2C19, 2D6, and 3A4 activity were concentration-dependent (Table 3), with IC_50_ values ranging from 0.79 to 108 M. Specifically, 78-TMF displayed significant inhibitory impacts on CYP1A2, CYP2C9, and CYP2C19, with IC_50_ values of 0.79 ± 0.12, 1.49 ± 0.16, and 1.85 ± 0.14, respectively. 3H7-TMF showed a significant inhibitory impact on CYP2D6 with an IC_50_ of 2.28 ± 0.46. The inhibitory effects of 3H7-TMF and 3H6-TMF on CYP3A4 were found to be strong, with IC_50_ values of 0.15 ± 0.07 and 0.44 ± 0.12, respectively.

#### 2.3.2. CYP Inhibitory Effect of TMF Structure

*Thyme* leaves (*Thymus saturoides*) contain a flavone known as 4′,5-dihydroxy-3′,6,7,8-tetramethoxy flavone (8-methoxycirsilineol), which exhibited potent inhibition against cytochrome P450s (CYP) 1A2 and 3A4 in vitro with IC_50_ values of 2.41 and 1.71 µM, respectively [32]. Recent molecular studies have shed light on the underlying cellular signaling pathways for the mechanism of action of flavonoids. Nobiletin (3′,4′,5,6,7,8-hexamethoxyflavone) boosts the cytostatic effect in (ER+) MCF-7 breast cancer cells by increasing the expression of inhibitors that selectively target cytochrome P450 family members, CYP1B1 and CYP1A1 [33,34]. Additionally, 5,7-dimethoxyflavone (5,7-DMF) and 3,5,7,3′,4′-pentamethoxyflavone (3,5,7,3′,4′-PMF) inhibits CYP3A, and this inhibition may be time-dependent but not irreversible [35]. Studies on the structure–affinity relationship have shown that 3-OH, 5-OH, 3′-OCH_3_, and 4′-OCH_3_ are crucial for flavonoids’ binding to P-gp [20]. The inhibition and induction of CYP3A4 and CYP1A2 enzymes can be attributed to nobiletin (3′,4′,5,6,7,8-Hexamethoxyflavone), sinensetin (3′,4′,5,6,7-Pentamethoxyflavone), and tangeretin (4′,5,6,7,8-Pentamethoxyflavone) [36]. The 3′ and 4′ positions on the B-ring of flavonoid structure are the primary sites of biotransformation. The number and position of the hydroxyl and methoxy groups on B-ring influence the metabolism significantly. Previous studies have shown that hesperetin, pinocembrin, chrysin, isorhamnetin, and morin have inhibitory effects on CYP1A2 activity. Similarly, apigenin, tangeretin, galangin, and isorhamnetin have been found to inhibit CYP2A6 activity, while chrysin, chrysin-dimethylether, and galangin have demonstrated inhibition of CYP2C8. These findings suggest that different forms of CYP enzymes may contribute to variations in the inhibitory activities of polymethoxylated flavones (PMFs) among individuals, potentially leading to differences in their biological effects [37]. We investigated the inhibition of three enzymes (CYP2C9, CYP2C19, and CYP1A2) by 78-TMF. For 57-TMF, its A ring lacked neighborhood methoxy groups, resulting in lower inhibition capability than other TMFs. 3H7-TMF and 3H6-TMF both had lower IC_50_ values due to the additional hydroxy groups in the C ring and the A ring spacing the third methoxy groups. Interestingly, the presence of a 3-hydroxyl group in TMFs decreased the inhibition of CYP3A4 while decreasing the inhibition of CYP1A2, CYP2C9, and CYP2C19 (Table 3). The observed inhibitory effects observed may be attributed to the steric hindrance caused by the hydroxyl group on the 3′C position and the methoxy group on the 2′C position. These structural features of the flavonoids could potentially interfere with the activity of the respective CYP enzymes, leading to inhibition of their function. The inhibition of CYP1A2, 2C9, and 2C19 by 78-TMF was dose-dependent, with IC_50_ values of 0.79–1.85 μM. The IC50 values of 78-TMF on CYP1A2, 2C9, and 2C19 inhibition were similar to the positive control when compared to established CYP inhibitors (ketoconazole and furafylline). The similarity between 3H7-TMF and 3H6-TMF groups suggested that different effect of inhibition was acting on CYP1A2, CYP2D6, CYP2C9, CYP2C19, and CYP3A4 by those TMF with 3-hydroxyl group and with/without nearby methoxy groups. Therefore, the present study revealed that methoxy at partition position on ring A, methoxy at position 2′C on ring B and hydroxy on ring C improve the TMF inhibition efficiency of CYP enzymes.

The potent inhibitory effects of flavonoids on specific CYP enzymes have been discussed in the literature. Investigations have been performed on the structure–affinity relationship, emphasizing the significance of hydroxyl and methoxy groups on the B-ring for flavonoid binding to P-gp, and identifying the varied inhibitory effects of flavonoids on CYP enzymes, indicating potential differences in their biological effects. The flavonol compound (fisetin) demonstrated pronounced inhibition of CYP2C8, surpassing its main *O*-methyl metabolite geraldol [38]. In this study, the comparison between hydroxylated and non-hydroxylated forms highlights the impact of the hydroxyl group on inhibitory capabilities and potential steric hindrance effects. Hydroxyl-containing TMFs, especially 3H7-TMF, demonstrate outstanding performance, suggesting a crucial role in food–drug interactions.

The present research underscores the significance of understanding the TMF impact on drug metabolism and absorption, particularly for individuals on medications metabolized by CYP enzymes. Specific polyphenols activate certain CYP450 isoforms, producing inhibitory intermediate metabolites that form multi-form complexes and lead to isoform inactivation. Despite the crucial role of the reactive intermediate metabolite in the mechanism-based inactivation of the CYP450 isoform, the exact reactive metabolite remains unknown, hindering the determination of competitive, non-competitive, or mechanism-based inhibitions. Precisely categorizing the inhibition type for polyphenols poses challenges due to their diverse structures, multiple interaction modes, extensive metabolism, concentration-dependent effects, and variations in assay conditions.

The flavonol compound fisetin demonstrated pronounced reversible and non-competitive inhibition of CYP2C8, surpassing its main metabolite geraldol. This specific inhibition was validated in the context of CYP2C8-catalyzed paclitaxel hydroxylation in pooled human liver microsomes, as investigated by Shrestha et al. in 2018 [38]. The study investigated the interaction of competitive and non-competitive enzymes, primarily those targeting pharmaceuticals, with additional polyphenolic compounds. Mechanism-based inhibition occurs when a substrate forms a reactive intermediate, establishing a stable enzyme–intermediate complex that irreversibly diminishes enzyme activity. In this process, the enzyme initiates the breakdown of the substrate for inhibition to progress. As more drug molecules undergo metabolism, an increasing number of stable complexes are formed in the active sites, resulting in a rise in inhibition over time until reaching a plateau. In the case of mixed inhibition, both competitive and non-competitive inhibition take place. Mixed inhibitors can bind simultaneously to both the heme iron atom (at the active site) and lipophilic regions of the protein (allosteric site). Typically, mixed inhibitors exhibit greater potency than competitive or non-competitive inhibitors. Notably, imidazole antifungals like ketoconazole and fluconazole demonstrate potent, mixed reversible inhibition of CYP3As, as explained by Deodhar et al. [39]. Given these complexities, no reaction rate test was included in this study, preventing the classification of CYP450 enzymes into their inhibitory types. Investigation of food–drug interactions would help identify potential interactions mediated by enzyme inhibition, aiding in the swift identification and mitigation of drug interactions, and thus reducing preventable drug-related issues.

## 3. Materials and Methods

### 3.1. Materials and Chemicals

The following compounds were obtained from Alfa Aesar (Heysham, UK): 3′,4′,7,8-TMF (78-TMF),3′,4′,5,7-TMF (57-TMF), 3-hydroxy-2′,4′,5′,7-TMF (3H7-TMF), and 3-hydroxy-2′,4′,5′,6-TMF (3H6-TMF). All other chemicals used were of analytical grade. Liquid chromatographic grade solvents and reagents were provided by Mallinckrodt Baker (Phillipsburg, NJ, USA). All preparations were made with triply deionized water (Millipore, Bedford, MA, USA). P450 Baculosomes^®^ (microsomes prepared from insect cells containing a single human P450 and P450 reductase), control Baculosomes^®^ (microsomes prepared from insect cells infected with a control virus), regeneration system (glucose-6-phosphate dehydrogenase and glucose–6–phosphate), NADP^+^, Vivid^®^ substrates, and assay buffers were obtained from Invitrogen Corp. (Madison, WI, USA). Corning provided 384 low–volume black polystyrene plates for the assays (Acton, MA, USA).

### 3.2. Analysis and Characterization of TMFs by LC-MS

TMFs were analyzed using an electrospray ionization ion trap mass spectrometer and LC-MS on a Thermo HPLC system (Thermo Scientific hybrid quadrupole-Orbitrap mass spectrometers; San Jose, CA, USA). Separation was performed using a YMC-UltraHT Hydrosphere C_18_ column (YMC, Tokyo, Japan) with 100% methanol (solvent A) and 0.1% formic acid as elution solvents (solvent B). The elution program was as follows (with a flow rate of 0.3 mL/min): 0–60 min, linear gradient from 10 to 100% A; 65 min, linear gradient from 100 to 10% A. Mass spectrometry parameters were set with a spray voltage of 3500 V for negative (or positive) mode, CE-Inject voltage of 3.8 kV, DE-Inject voltage of 40V, sheath gas flow rate of 10 au, capillary temperature of 320 °C, and Z-lens voltage of 230 V. The mass of LC-MS product ions was calculated using the instrument’s second analyzer after the collision-activated dissociation of selected precursor ions in the collision cell of the Orbitrap mass spectrometer. Four transitions were monitored for each type of TMF (Table 1). LC-MS data were processed using Xcalibur version 4.3 data acquisition software. The coefficients of intraday and interday testing variation were 5% in the concentration range of 0.5–20.0 μg/mL. The lower limits of quantification for 78-TMF, 57-TMF, 3H7-TMF, and 3H6-TMF were 0.2, 0.3, 0.1, and 0.3 μg/mL, respectively, while the detection limits were 0.05, 0.07, 0.03, and 0.04 μg/mL. Part of the experiment was conducted at the National Pingtung University of Science and Technology.

#### Method Validation

The method’s linearity, accuracy, and precision were assessed. An external standard stock solution of 78-TMF, 57-TMF, 3H7-TMF, and 3H6-TMF (0.02–25.0 μg/mL) was prepared using methanol. Linearity was tested by successive dilutions from the stock solution, and a standard curve was constructed using a linear least-square regression equation generated from the peak area. The technique’s inter- and intraday variability were assessed at seven concentrations on the same day (six replications) and over six subsequent days. Precision, evaluated by the relative standard deviation (RSD), and accuracy (% bias) were calculated from observed concentrations. Intra-assay and inter-assay accuracy of 78-TMF, 57-TMF, 3H7-TMF, and 3H6-TMF levels fell well within predefined limits of acceptability. The accuracy of 78-TMF, 57-TMF, 3H7-TMF, and 3H6-TMF levels within both intra-assay and inter-assay assessments met predefined acceptability limits. Precision was maintained at less than 5% coefficient of variation (CV), and accuracy demonstrated a bias of 3%.

### 3.3. Caco-2 Cell Cultures

Caco-2 cells were cultured in Dulbecco’s modified Eagle’s medium (DMEM; GIBCO, Carlsbad, CA, USA) supplemented with 10% fetal bovine serum (GIBCO), 1% nonessential amino acid solution, and antibiotics (100 U/mL penicillin and 100 g/mL streptomycin from GIBCO) with a minor modification according to Lu [40]. Cells were grown in an incubator with 5% CO_2_ and 95% humidity at 37 °C, with medium replaced every 2–3 days. The tests were carried out using cells from passages 25–35. Transwell inserts with a polycarbonate membrane (diameter 1.2 cm, pore size 0.4 μm; Corning Costar Co., Corning, NY, USA) were used in 12-well plates. Caco-2 cells were planted at 5 × 10^5^ cells/cm^2^ on the membrane inserts, and 0.5 and 1.5 mL of culture media were placed on the apical and basolateral sides, respectively. The medium was changed three times a week, and the Caco-2 cells used in the studies had been cultured in the Transwells for approximately 21 days. The integrity of the cell layer was assessed using Millicell ERS equipment by measuring transepithelial electrical resistance (TEER) values (Millipore Co., Bedford, MA, USA). Monolayers with TEER values greater than 400 Ωcm^2^ were used for transport and absorption studies.

### 3.4. Transport Experiments

Transport experiments were conducted using modified methodologies from the literature [41,42]. Before adding the test solution, the apical and basolateral sides of the Coco-2 cell monolayer grown in Transwell inserts were washed three times with Hank’s balanced salt solution (GIBCO). The apical-to-basolateral permeability was measured by adding 0.5 mL of the test solution containing 300 μg/mL TMFs and 1.5 mL of HBSS to the apical and basolateral sides, respectively, and incubating at 37 °C for 0.5, 1, 2, and 4 h. The concentration of TMF utilized was determined using the Caco-2 cell viability test findings. TMF was dissolved and diluted with HBSS before the experiment began. The apical and basolateral solutions were collected after incubation for 0.5, 1, 2, and 4 h, and the concentration of TMF was determined using LC-MS. The apparent permeability coefficient (*P*_app_ in cm/s) was calculated over a four-hour duration using Equation (1):

*P*_app_ = (*V*/*AC*_0_) × (*dC*/*dt*) (1)

where *V* is the volume of solution on the basolateral side (1.5 mL); *A* is the membrane surface area (1.12 cm^2^); *C*_0_ is the initial concentration on the apical side; and *dC*/*dt* is the change in concentration in the basolateral side over time.

The transport efficiency (%) was calculated using Equation (2):

*Transport efficiency* (%) = (*Cbs*)/(*Cas*) × 100 (%) (2)

where *Cbs* is the concentration of TMF transported in the basolateral side; and *Cas* is the initial concentration of TMF added to the apical side.

### 3.5. Absorption Experiments

The absorption investigation was carried out with certain modifications based on published methodologies [41]. The cell membranes were rinsed three times with HBSS before being withdrawn from the inserts following media collection from the apical and basolateral sides. Methanol was used to extract TMF. Cells were sonicated for 15 min and centrifuged for 5 min at 4 °C at 2000× *g*. The methanol supernatant was collected, and the cells were extracted twice with 1 mL of methanol, vortexed for 1 min, then centrifuged at 2000× *g* for 5 min at 4 °C. The supernatant was evaporated to dryness under nitrogen and reconstituted in 500 μL of methanol. The resulting extract solutions were injected into the LC-MS.

### 3.6. CYP Inhibition Assay

Five μL of 3× P450 Baculosomes^®^/Vivid^®^ Substrate or 3× control Baculosomes^®^/Vivid^®^ Substrate (Thermo Scientific, Waltham, MA, USA) in assay buffer (0.2× for CYP1A2, 2C9, 2C19, 2D6, and 3A4 tests) were distributed on the assay plates. Each TMF was tested in triplicate under two conditions: in the presence of P450 to determine inhibition and in the absence of P450 to detect autofluorescent chemicals. In the presence of P450 (*n* = 16), 1% DMSO was used in the control wells to mimic 100% activity, while in the absence of P450 (*n* = 16), it represented 0% activity. After incubating the plates for 10 min, 5 μL of 3× Regeneration System/NADP^+^ was added to each well. The plates were then shaken for 30 s and incubated for 25 min as described previously. The reaction was halted by adding five μL of 0.5 M tris base (pH 10.5) to all wells and shaking for 30 s. The plates were then read using the fluorescent excitation and emission parameters given on a Safire plate reader (Tecan, Durham, NC, USA) [43].

### 3.7. IC_50_ Determination of CYP

TMFs were assessed using a 10-dose IC_50_ method, involving threefold successive dilutions starting at 100 μM. Additionally, a 10-dose IC_50_ mode was implemented for ketoconazole and furafylline, using five-fold serial dilutions starting at 20 μM as positive controls. Ketoconazole and furafylline were positive controls in cytochrome P450 (CYP450) inhibition tests due to their well-established potency as inhibitors of specific CYP isoforms, providing a baseline for assessing the inhibitory effects of new compounds and ensuring reliability in evaluating potential drug interactions and adverse effects on CYP enzymes. The percent activity of sample wells was plotted against the logarithmic value of each test drug concentration, creating a 10-point dose–response curve. IC_50_ values were then calculated based on the curves of mean enzyme activity versus inhibitor concentration. The Quest Graph™ IC_50_ Calculator from AAT Bioquest, Inc. (Bengaluru, India) was utilized for these computations.

### 3.8. Statistical Analysis

All the samples were extracted three times. TMF concentrations (78-TMF, 57-TMF, 3H7-TMF, and 3H6-TMF) were measured in μg/mL medium. Before analysis, the medium from six wells was combined. The data were analyzed using analysis of variance (ANOVA), and statistically significant differences were considered at *p* < 0.05.

## 4. Conclusions

The findings of this study indicate that the structure of TMFs may influence the permeability of intestinal cells and the inhibition of CYP enzymes. 3H7-TMF exhibited faster transport and absorption efficiency in the Caco-2 cell monolayer model than other TMFs. As a hydrophilic substance, dissolved hydroxyl-TMF can readily permeate the gastrointestinal tract and achieve high concentration in the gut. TMFs, including 78-TMF, 57-TMF, 3H7-TMF, and 3H6-TMF, exhibited potent inhibitory effects on CYP, which could lead to abnormal absorption patterns by interfering with phase I metabolism in the gut or liver. 3H7-TMF, which contains hydroxyl groups and methoxy at position 7C on ring A, showed improved membrane permeability and solubility due to steric hindrance from hydroxyl bonds. It also inhibited CYP3A4 more effectively. The study suggests that TMFs play a crucial role in food–drug interactions, and the exceptional performance of hydroxyl-containing TMFs could be attributed to their hydrophilic nature, which provides a larger surface area and improves mucosal permeability and solubility, leading to enhanced intestinal absorption. Moreover, this study showed for the first time that TMFs exhibit strong to moderate inhibitory capabilities against CYP. Citrus fruits are renowned for their established nutraceutical and medicinal benefits, providing protection against a range of chronic diseases. This protection is attributed in part to their abundant nutrients with various physiological activities. For individuals taking medications metabolized by CYP enzymes, it is recommended to avoid concurrently consuming supplements containing TMFs to mitigate the risk of potential adverse events stemming from interactions between food and drugs.

## Figures and Tables

**Table 1:** LC-MS detection of TMF.

TMF	LC-MS Parent Ion Selection	Fragment Ions, Positively Charged	Chemical Formula	Molecular Weight/ Exact Mass
78-TMF	343.12 [M + H]^+^	327.09, 312.06, 299.09, 164.08	C_19_H_18_O_6_	342.35/342.11
57-TMF	343.12 [M + H]^+^	327.09, 312.06, 299.09, 167.03	C_19_H_18_O_6_	342.35/342.11
3H7-TMF	359.1 [M + H]^+^	344.09, 329.06, 299.09, 168.08, 151.04	C_19_H_18_O_7_	358.35/358.11
3H6-TMF	359.1 [M + H]^+^	344.09, 329.06, 301.07, 273.08, 151.04	C_19_H_18_O_7_	358.35/358.11

**Table 2:** Transport efficiency (%) of TMFs across Caco-2 cell monolayer from apical to basolateral side.

Time (h)	Transport Efficiency (%) *			
	78-TMF	57-TMF	3H7-TMF	3H6-TMF
1	30.49 ± 0.05 ^c^**	22.48 ± 3.90 ^c^	37.82 ± 0.07 ^c^	25.42 ± 1.92 ^c^
2	33.98 ± 0.75 ^b^	33.03 ± 0.46 ^b^	41.96 ± 1.14 ^b^	33.27 ± 2.59 ^b^
4	37.58 ± 2.11 ^a^	34.00 ± 1.81 ^ab^	46.29 ± 2.16 ^a^	39.43 ± 3.50 ^a^

* Transport efficiency (%) = (TMFs concentration on basolateral side)/(initial TMFs concentration loaded on apical side) × 100. ** Data are expressed as mean ± S.D and different letters indicate significant differences at *p* < 0.05.

**Table 3:** Cytochrome P450 inhibitory activities (IC_50_, μM) of TMF.

	IC_50_(μM) *	CYP1A2	CYP2D6	CYP2C9	CYP2C19	CYP3A4
TMF						
78-TMF		0.79 ± 0.12 ^c^	ND	1.49 ±0.16 ^c^	1.85 ±0.14 ^b^	108 ± 6.31 ^a^
57-TMF		35.6 ± 12.31 ^a^	ND	62.5 ± 2.12 ^a^	ND	3.56 ± 1.23 ^b^
3H7-TMF		15.5 ±1.95 ^b^	2.28 ± 0.46 ^b^	18.3 ± 8.93 ^b^	14.8 ± 3.15 ^a^	0.15 ± 0.07 ^c^
3H6-TMF		20.6 ± 0.98 ^a^	95.5 ± 3.21 ^a^	8.03 ±3.75 ^b^	10.2 ± 2.47 ^a^	0.44 ± 0.12 ^c^
Ketoconazole		ND	2.35 ± 0.46 ^b^	1.62 ± 0.74 ^c^	1.31 ± 0.47 ^b^	0.022 ± 0.013 ^d^
Furafylline		0.72 ± 0.24 ^c^	ND	ND	ND	ND

* Data are expressed as mean ± S.D. Data in the same row with different letters are significantly different at *p* < 0.05. ND: Not Detected.

## Data Availability

Data are available on request from the authors.

## Supplementary Materials

The following supporting information can be downloaded at: https://www.mdpi.com/article/10.3390/molecules29020322/s1, Table S1: the occurrence of 3′,4′,5,7-tetramethoxyflavone in natural products; Table S2: chemical–disease co-occurrences that can be prevented by 5,7,3′,4′-tetramethoxyflavone. Refs. [44,45,46,47,48,49,50,51,52,53,54,55,56,57,58,59,60,61,62,63,64,65,66,67,68,69,70] are cited in the Supplementary Materials.

## Abbreviations

PMFs: polymethoxyflavones; TMF: tetramethoxyflavone; 78-TMF: 3′,4′,7,8-tetramethoxyflavone; 57-TMF, 3′,4′,5,7-tetramethoxyflavone; 3H7-TMF: 3-hydroxy-2′,4′,5′,7-tetramethoxyflavone; 3H6-TMF: 3-hydroxy-2′,4′,5′,6-tetramethoxyflavone; *P*_app_: apparent permeability; CYP 450: cytochrome P450. **Chemical Compounds:** 3′,4′,7,8-tetramethoxyflavone (PubChem CID: 4033898); 3′,4′,5,7-tetramethoxyflavone (PubChem CID: 631170); 3-hydroxy-2′,4′,5′,7-tetramethoxyflavone (PubChem CID: 24721676); 3-hydroxy-2′,4′,5′,6-tetramethoxyflavone (PubChem CID: 24721087).

## References

[1] Maqbool Z., Khalid W., Atiq H.T., Koraqi H., Javaid Z., Alhag S.K., Al-Shuraym L.A., Bader D.M.D., Almarzuq M., Afifi M. (2023). Citrus Waste as Source of Bioactive Compounds: Extraction and Utilization in Health and Food Industry. Molecules.

[2] Yu Q., Tao Y., Huang Y., Zogona D., Wu T., Liu R., Pan S., Xu X. (2022). Aged Pericarpium Citri Reticulatae ‘Chachi’ Attenuates Oxidative Damage Induced by tert-Butyl Hydroperoxide (t-BHP) in HepG2 Cells. Foods.

[3] Wang Y., Mei X., Liu Z., Li J., Zhang X., Lang S., Dai L., Zhang J. (2019). Drug Metabolite Cluster-Based Data-Mining Method for Comprehensive Metabolism Study of 5-hydroxy-6,7,3′,4′-tetramethoxyflavone in Rats. Molecules.

[4] Xu Y., Lv X., Yang G., Zhan J., Li M., Long T., Ho C.T., Li S. (2019). Simultaneous separation of six pure polymethoxyflavones from sweet orange peel extract by high performance counter current chromatography. Food Chem..

[5] Qiu M., Wei W., Zhang J., Wang H., Bai Y., Guo D. (2023). A Scientometric Study to a Critical Review on Promising Anticancer and Neuroprotective Compounds: Citrus Flavonoids. Antioxidants.

[6] Mushtaq Z., Aslam M., Imran M., Abdelgawad M.A., Saeed F., Khursheed T., Umar M., Al Abdulmonem W., Al Ghorab A.H., Alsagaby S.A. (2023). Polymethoxyflavones: An updated review on pharmacological properties and underlying molecular mechanisms. Int. J. Food Propert..

[7] Yang J., Liu H., Li L., Liu H., Shi W., Wu L. (2015). The chondroprotective role of TMF in PGE2-induced apoptosis associating with endoplasmic reticulum stress. Evid.-Based Complem. Altern. Med..

[8] Ahmed O.M., AbouZid S.F., Ahmed N.A., Zaky M.Y., Liu H. (2021). An Up-to-Date Review on Citrus Flavonoids: Chemistry and Benefits in Health and Diseases. Curr. Pharm. Des..

[9] Wu L., Liu H., Li L., Liu H., Yang K., Liu Z., Huang H. (2014). 5,7,3′,4′-Tetramethoxyflavone exhibits chondroprotective activity by targeting beta-catenin signaling in vivo and in vitro. Biochem. Biophys. Res. Commun..

[10] Peng F., Huang X., Shi W., Xiao Y., Jin Q., Li L., Xu D., Wu L. (2021). 5,7,3′,4′-tetramethoxyflavone ameliorates cholesterol dysregulation by mediating SIRT1/FOXO3a/ABCA1 signaling in osteoarthritis chondrocytes. Future Med. Chem..

[11] Wu L., Liu H., Li L., Xu D., Gao Y., Guan Y., Chen Q. (2018). 5,7,3′,4′-Tetramethoxyflavone protects chondrocytes from ER stress-induced apoptosis through regulation of the IRE1alpha pathway. Connect. Tissue Res..

[12] Yang G., Xia X., Zhong H., Shen J., Li S. (2021). Protective Effect of Tangeretin and 5-Hydroxy-6,7,8,3′,4′-Pentamethoxyflavone on Collagen-Induced Arthritis by Inhibiting Autophagy via Activation of the ROS-AKT/mTOR Signaling Pathway. J. Agric. Food Chem..

[13] Tan C.S., Yam M.F. (2018). Mechanism of vasorelaxation induced by 3′-hydroxy-5,6,7,4′-tetramethoxyflavone in the rats aortic ring assay. Naunyn-Schmiedeberg’s Arch. Pharmacol..

[14] Ashrafizadeh M., Zarrabi A., Saberifar S., Hashemi F., Hushmandi K., Hashemi F., Moghadam E.R., Mohammadinejad R., Najafi M., Garg M. (2020). Nobiletin in Cancer Therapy: How This Plant Derived-Natural Compound Targets Various Oncogene and Onco-Suppressor Pathways. Biomedicines.

[15] Yang G., Lin C.C., Yang Y., Yuan L., Wang P., Wen X., Pan M.H., Zhao H., Ho C.T., Li S. (2019). Nobiletin Prevents Trimethylamine Oxide-Induced Vascular Inflammation via Inhibition of the NF-κB/MAPK Pathways. J. Agric. Food Chem..

[16] Lai C.S., Wu J.C., Ho C.T., Pan M.H. (2015). Disease chemopreventive effects and molecular mechanisms of hydroxylated polymethoxyflavones. Biofactors.

[17] Fontana G., Bruno M., Sottile F., Badalamenti N. (2022). The Chemistry and the Anti-Inflammatory Activity of Polymethoxyflavonoids from *Citrus* Genus. Antioxidants.

[18] Zhao M., Ma J., Li M., Zhang Y., Jiang B., Zhao X., Huai C., Shen L., Zhang N., He L. (2021). Cytochrome P450 enzymes and drug metabolism in humans. Int. J. Mol. Sci..

[19] Manikandan P., Nagini S. (2018). Cytochrome P450 structure, function and clinical significance: A review. Curr. Cancer Drug Targets.

[20] Fang Y., Xia M., Liang F., Cao W., Pan S., Xu X. (2019). Establishment and use of human mouth epidermal carcinoma (KB) cells overexpressing P-glycoprotein to characterize structure requirements for flavonoids transported by the efflux transporter. J. Agric. Food Chem..

[21] You Q., Li D., Ding H., Chen H., Hu Y., Liu Y. (2021). Pharmacokinetics and Metabolites of 12 Bioactive Polymethoxyflavones in Rat Plasma. J. Agric. Food Chem..

[22] Iftikhar M., Iftikhar A., Zhang H., Gong L., Wang J. (2020). Transport, metabolism and remedial potential of functional food extracts (FFEs) in Caco-2 cells monolayer: A review. Food Res. Int..

[23] Naseem A., Pal A., Gowan S., Asad Y., Donovan A., Temesszentandrási-Ambrus C., Kis E., Gaborik Z., Bhalay G., Raynaud F. (2022). Intracellular Metabolomics Identifies Efflux Transporter Inhibitors in a Routine Caco-2 Cell Permeability Assay-Biological Implications. Cells.

[24] Berben P., Bauer-Brandl A., Brandl M., Faller B., Flaten G.E., Jacobsen A.C., Brouwers J., Augustijns P. (2018). Drug permeability profiling using cell-free permeation tools: Overview and applications. Eur. J. Pharm. Sci..

[25] He S., Zhiti A., Barba-Bon A., Hennig A., Nau W.M. (2020). Real-Time Parallel Artificial Membrane Permeability Assay Based on Supramolecular Fluorescent Artificial Receptors. Front. Chem..

[26] Tsuchitani T., Akiyoshi T., Imaoka A., Ohtani H. (2022). Mechanistic bottom-up estimation of passive drug absorption from the gastrointestinal tract: Comparison among primary cultured human intestinal cells, Caco-2 cells, artificial membrane, and animal scale-up. Int. J. Clin. Pharmacol. Ther..

[27] Pham-The H., Cabrera-Pérez M.Á., Nam N.H., Castillo-Garit J.A., Rasulev B., Le-Thi-Thu H., Casañola-Martin G.M. (2018). In Silico Assessment of ADME Properties: Advances in Caco-2 Cell Monolayer Permeability Modeling. Curr. Top. Med. Chem..

[28] Li S., Pan M.H., Wang Z., Lambros T., Ho C.T. (2008). Biological activity, metabolism and separation of polymethoxyflavonoids from citrus peels. Tree For. Sci. Biotechnol..

[29] Li S., Pan M.H., Lo C.Y., Tan D., Wang Y., Shahidi F., Ho C.T. (2009). Chemistry and health effects of polymethoxyflavones and hydroxylated polymethoxyflavones. J. Funct. Foods.

[30] Verma D., Mitra D., Paul M., Chaudhary P., Kamboj A., Thatoi H., Janmeda P., Jain D., Panneerselvam P., Shrivastav R. (2021). Potential inhibitors of SARS-CoV-2 (COVID 19) proteases PLpro and Mpro/3CLpro: Molecular docking and simulation studies of three pertinent medicinal plant natural components. Curr. Res. Pharmacol. Drug Discov..

[31] Nagayoshi H., Murayama N., Takenaka S., Kim V., Kim D., Komori M., Yamazaki H., Guengerich F.P., Shimada T. (2021). Roles of cytochrome P450 2A6 in the oxidation of flavone, 4′-hydroxyflavone, and 4′-, 3′-, and 2′-methoxyflavones by human liver microsomes. Xenobiotica.

[32] Brahmi Z., Niwa H., Yamasato M., Shigeto S., Kusakari Y., Sugaya K., Onose J., Abe N. (2011). Effective cytochrome P450 (CYP) inhibitor isolated from thyme (*Thymus saturoides*) purchased from a Japanese market. Biosci. Biotechnol. Biochem..

[33] Surichan S., Androutsopoulos V.P., Sifakis S., Koutala E., Tsatsakis A., Arroo R.R., Boarder M.R. (2012). Bioactivation of the citrus flavonoid nobiletin by CYP1 enzymes in MCF7 breast adenocarcinoma cells. Food Chem. Toxicol..

[34] Koolaji N., Shammugasamy B., Schindeler A., Dong Q., Dehghani F., Valtchev P. (2020). Citrus Peel Flavonoids as Potential Cancer Prevention Agents. Curr. Dev. Nutr..

[35] Kashiwabuchi Y., Nishimura Y., Kurata N., Iwase M., Kiuchi Y., Nobe K. (2022). Inhibition of CYP3A-mediated Midazolam Metabolism by *Kaempferia parviflora*. Food Saf..

[36] Weiss J., Gattuso G., Barreca D., Haefeli W.E. (2020). Nobiletin, sinensetin, and tangeretin are the main perpetrators in clementines provoking food-drug interactions in vitro. Food Chem..

[37] Bojić M., Kondža M., Rimac H., Benković G., Maleš Ž. (2019). The Effect of Flavonoid Aglycones on the CYP1A2, CYP2A6, CYP2C8 and CYP2D6 Enzymes Activity. Molecules.

[38] Shrestha R., Kim J.H., Nam W., Lee H.S., Lee J.M., Lee S. (2018). Selective inhibition of CYP2C8 by fisetin and its methylated metabolite, geraldol, in human liver microsomes. Drug Metab. Pharmacokinet..

[39] Deodhar M., Al Rihani S.B., Arwood M.J., Darakjian L., Dow P., Turgeon J., Michaud V. (2020). Mechanisms of CYP450 Inhibition: Understanding Drug-Drug Interactions Due to Mechanism-Based Inhibition in Clinical Practice. Pharmaceutics.

[40] Lu C., Fu K., Cao K., Wei J., Zhou J., Zhao D., Li N., Lu Y., Chen X., Zhang Y. (2020). Permeability and transport mechanism of trihexyphenidyl hydrochloride in Caco-2 cell monolayer model with a validated UPLC-MS method. J. Pharm. Biomed. Anal..

[41] Inada A., Sawao A., Takahashi K., Oshima T. (2022). Enhanced water dispersibility and Caco-2 cell monolayer permeability of quercetin by complexation with casein hydrolysate. J. Food Sci..

[42] Lanevskij K., Didziapetris R. (2019). Physicochemical QSAR Analysis of Passive Permeability Across Caco-2 Monolayers. J. Pharm. Sci..

[43] Jan K.C., Chang Y.W., Hwang L.S., Ho C.T. (2012). Tissue distribution and cytochrome P450 inhibition of sesaminol and its tetrahydrofuranoid metabolites. J. Agric. Food Chem..

[44] Fraser A.W., Lewis J.R. (1974). Eupatorin, a constituent of *Merrillia caloxylon*. Planta Med..

[45] Azuma T., Tanaka Y., Kikuzaki H. (2008). Phenolic glycosides from *Kaempferia parviflora*. Phytochemistry.

[46] Chaipech S., Morikawa T., Ninomiya K., Yoshikawa M., Pongpiriyadacha Y., Hayakawa T., Muraoka O. (2012). Structures of two new phenolic glycosides, kaempferiaosides A and B, and hepatoprotective constituents from the rhizomes of *Kaempferia parviflora*. Chem. Pharm. Bull..

[47] Mizuno M., Iinuma M., Ohara M., Tanaka T., Iwamasa M. (1991). Chemotaxonomy of the Genus Citrus Based on Polymethoxyflavones. Chem. Pharm. Bull..

[48] Zhang J.Y., Li N., Che Y.Y., Zhang Y., Liang S.X., Zhao M.B., Jiang Y., Tu P.F. (2011). Characterization of seventy polymethoxylated flavonoids (PMFs) in the leaves of *Murraya paniculata* by on-line high-performance liquid chromatography coupled to photodiode array detection and electrospray tandem mass spectrometry. J. Pharm. Biomed. Anal..

[49] Tuchinda P., Reutrakul V., Claeson P., Pongprayoon U., Sematongz T., Santisuk T., Taylor W.C. (2002). Anti-inflammatory cyclohexenyl chalcone derivatives in Boesenbergia pandurata. Phytochemistry.

[50] Jaipetch T., Reutrakul V., Tuntiwachwuttikul P., Santisuk T. (1983). Flavonoids in the black rhizomes of Boesenbergia panduta. Phytochemistry.

[51] Asamenew G., Kim H.W., Lee M.K. (2019). Characterization of phenolic compounds from normal ginger (Zingiber officinale Rosc.) and black ginger (*Kaempferia parviflora* Wall.) using UPLC–DAD–QToF–MS. Eur. Food Res. Technol..

[52] Rajudin E., Ahmad F., Sirat H.M., Arbain D., Aboul-Enein H.Y. (2010). Chemical constituents from tiger’s betel, Piper porphyrophyllum N.E.Br. (*Fam. Piperaceae*). Nat. Prod. Res..

[53] Kim Y.D., Ko W.J., Koh K.S., Jeon Y.J., Kim S.H. (2009). Composition of Flavonoids and Antioxidative Activity from Juice of Jeju Native Citrus Fruits during Maturation. Korean J. Nutr..

[54] Lu W.C., Sheen J.F., Hwang L.S., Wei G.J. (2012). Identification of 5,7,3′,4′-tetramethoxyflavone metabolites in rat urine by the isotope-labeling method and ultrahigh-performance liquid chromatography-electrospray ionization-mass spectrometry. J. Agric. Food Chem..

[55] Fourie T.G., du Preez I.C., Roux D.G. (1972). 3′,4′,7,8-tetrahydroxyflavonoids from the heartwood of *Acacia nigrescens* and their conversion products. Phytochemistry.

[56] Li S.M., Yang J.L., Liu Y.P., Fu Y.H. (2015). Studies on non-alkaloid constituents from *Alstonia mairei*. Chin. Tradit. Herb. Drugs.

[57] Ballesteros J.F., Sanz M.J., Ubeda A., Miranda M.A., Iborra S., Payá M., Alcaraz M.J. (1995). Synthesis and pharmacological evaluation of 2′-hydroxychalcones and flavones as inhibitors of inflammatory mediators generation. J. Med. Chem..

[58] Weng Z., Patel A.B., Panagiotidou S., Theoharides T.C. (2015). The novel flavone tetramethoxyluteolin is a potent inhibitor of human mast cells. J. Allergy Clin. Immunol..

[59] Bawazeer M.A., Theoharides T.C. (2019). IL-33 stimulates human mast cell release of CCL5 and CCL2 via MAPK and NF-κB, inhibited by methoxyluteolin. Eur. J. Pharmacol..

[60] Patel A.B., Theoharides T.C. (2017). Methoxyluteolin Inhibits Neuropeptide-stimulated Proinflammatory Mediator Release via mTOR Activation from Human Mast Cells. J. Pharmacol. Exp. Ther..

[61] Taracanova A., Alevizos M., Karagkouni A., Weng Z., Norwitz E., Conti P., Leeman S.E., Theoharides T.C. (2017). SP and IL-33 together markedly enhance TNF synthesis and secretion from human mast cells mediated by the interaction of their receptors. Proc. Natl. Acad. Sci. USA.

[62] Theoharides T.C., Tsilioni I. (2018). Tetramethoxyluteolin for the Treatment of Neurodegenerative Diseases. Curr. Top. Med. Chem..

[63] Theoharides T.C., Stewart J.M., Tsilioni I. (2017). Tolerability and benefit of a tetramethoxyluteolin-containing skin lotion. Int. J. Immunopathol. Pharmacol..

[64] Huang X., Chen Z., Shi W., Zhang R., Li L., Liu H., Wu L. (2019). TMF inhibits miR-29a/Wnt/β-catenin signaling through upregulating Foxo3a activity in osteoarthritis chondrocytes. Drug Des. Devel Ther..

[65] Yuan X., Li L., Shi W., Liu H., Huang X., Liu Z., Wu L. (2017). TMF protects chondrocytes from ER stress-induced apoptosis by down-regulating GSK-3β. Biomed. Pharmacother..

[66] Patel A.B., Tsilioni I., Weng Z., Theoharides T.C. (2018). TNF stimulates IL-6, CXCL8 and VEGF secretion from human keratinocytes via activation of mTOR, inhibited by tetramethoxyluteolin. Exp. Dermatol..

[67] Sae-Wong C., Matsuda H., Tewtrakul S., Tansakul P., Nakamura S., Nomura Y., Yoshikawa M. (2011). Suppressive effects of methoxyflavonoids isolated from *Kaempferia parviflora* on inducible nitric oxide synthase (iNOS) expression in RAW 264.7 cells. J. Ethnopharmacol..

[68] Theoharides T.C., Tsilioni I. (2020). Amyotrophic Lateral Sclerosis, Neuroinflammation, and Cromolyn. Clin. Ther..

[69] Nakao K., Murata K., Deguchiz T., Itoh K., Fujita T., Higashino M., Yoshioka Y., Matsumura S., Tanaka R., Shinada T. (2011). Xanthine oxidase inhibitory activities and crystal structures of methoxyflavones from *Kaempferia parviflora* rhizome. Biol. Pharm. Bull..

[70] Murata K., Hayashi H., Matsumura S., Matsuda H. (2013). Suppression of benign prostate hyperplasia by *Kaempferia parviflora* rhizome. Pharmacogn. Res..

