# Supplementary material for: Hydroxylated Tetramethoxyflavone Affects Intestinal Cell Permeability and Inhibits Cytochrome P450 Enzymes
Source: Molecules. 2024 Jan 9;29(2):322. doi: 10.3390/molecules29020322 (PMC10820070; doi:10.3390/molecules29020322)
Supplement: Supplementary file 1 [file molecules-29-00322-s001.zip › molecules-2783340-supplementary.pdf]

Table S1. The occurrence of 3’, 4’, 5, 7-tetramethoxyflavone in natural products.

Plant common name	Scientific name	Form of tetramethoxyflavone	Reference
Knobthorn Acacia	*Acacia nigrescens*	3’, 4’, 5, 7-tetramethoxyflavone	Fourie *et al.,* 1972
Maire's Alstonia	*Alstonia mairei*	3’, 4’, 5, 7-tetramethoxyflavone	KNApSAcK Species-Metabolite Database
Java Tea	*Orthosiphon spicatus*	3’, 4’, 5, 7-tetramethoxyflavone	KNApSAcK Species-Metabolite Database
Redwood	*Merrillia caloxylon*	3’, 4’, 5, 7- tetramethoxyflavone	Fraser *et al.,* 1974
Fingerroot	*Boesenbergia pandurata*	3’, 4’, 5, 7- tetramethoxyflavone	KNApSAcK Species-Metabolite Database
Akane Madder	*Rubia akane*	3’, 4’, 5, 7-tetramethoxyflavone	Natural Product Activity and Species Source (NPASS)
	Senecio platyphylloides	3’, 4’, 5, 7- tetramethoxyflavone	Natural Product Activity and Species Source (NPASS)
Circassian Milk Vetch	*Astragalus circassicus*	3’, 4’, 5, 7- tetramethoxyflavone	Natural Product Activity and Species Source (NPASS)
	Erythrina hypaphorus	3’, 4’, 5, 7- tetramethoxyflavone	Natural Product Activity and Species Source (NPASS)
	Sphaeria rousseliana	3’, 4’, 5, 7- tetramethoxyflavone	Natural Product Activity and Species Source (NPASS)
Fragrant Lip Fern	*Cheilanthes fragrans*	3’, 4’, 5, 7- tetramethoxyflavone	Natural Product Activity and Species Source (NPASS)
Kahikatea	*Podocarpus dacrydioides*	3’, 4’, 5, 7- tetramethoxyflavone	Natural Product Activity and Species Source (NPASS)
Hellebore	*Helleborus macranthus*	3’, 4’, 5, 7- tetramethoxyflavone	Natural Product Activity and Species Source (NPASS)
Rosemary Willow	*Salix rosmarinifolia*	3’, 4’, 5, 7- tetramethoxyflavone	Natural Product Activity and Species Source (NPASS)
	Aniba santalodora	3’, 4’, 5, 7- tetramethoxyflavone	Natural Product Activity and Species Source (NPASS)

	Colchicum filifolium	3’, 4’, 5, 7- tetramethoxyflavone	Natural Product Activity and Species Source (NPASS)
Kashmir Iris	*Iris kashmiriana*	3’, 4’, 5, 7- tetramethoxyflavone	Natural Product Activity and Species Source (NPASS)
Velvet Belly Lanternshark	*Etmopterus spinax (velvet belly lantern shark)*	3’, 4’, 5, 7- tetramethoxyflavone	Natural Product Activity and Species Source (NPASS)
	Lemonia spectabilis	3’, 4’, 5, 7- tetramethoxyflavone	Natural Product Activity and Species Source (NPASS)
Joseph Scale	*Matsucoccus josephi*	3’, 4’, 5, 7- tetramethoxyflavone	Natural Product Activity and Species Source (NPASS)
	Centaurea sonchifolia	3’, 4’, 5, 7- tetramethoxyflavone	Natural Product Activity and Species Source (NPASS)
Sand Chamomile	*Matricaria sabulosa*	3’, 4’, 5, 7- tetramethoxyflavone	Natural Product Activity and Species Source (NPASS)
Thai Black Ginger	*Kaempferia parviflora*	3’, 4’, 5, 7- tetramethoxyflavone	Azuma *et al.,* 2008, Chaipech *et al.,* 2012, Lu *et al.,* 2012<br>KNApSAcK Species-Metabolite Database<br>LOTUS - the natural products occurrence database
Giant Sensitive Plant	*Mimosa pigra*	3’, 4’, 5, 7- tetramethoxyflavone	Natural Product Activity and Species Source (NPASS)
Konjac	*Amorphophallus konjac*	3’, 4’, 5, 7- tetramethoxyflavone	Natural Product Activity and Species Source (NPASS)
	Lonchocarpus chiricanus	3’, 4’, 5, 7- tetramethoxyflavone	Natural Product Activity and Species Source (NPASS)
Cat's Whiskers	*Orthosiphon aristatus*	3’, 4’, 5, 7- tetramethoxyflavone	LOTUS - the natural products occurrence database
	Mucuna holtonii	3’, 4’, 5, 7- tetramethoxyflavone	Natural Product Activity and Species Source (NPASS)
Canary Island Pheasant's Eye	*Bethencourtia palmensis*	3’, 4’, 5, 7- tetramethoxyflavone	Natural Product Activity and Species Source (NPASS)
Kumquat	*Citrus japonica*	3’, 4’, 5, 7- tetramethoxyflavone	Mizuno *et al.,* 1991<br>LOTUS - the natural products occurrence database
Orange Jessamine	*Murraya paniculata*	3’, 4’, 5, 7- tetramethoxyflavone	LOTUS - the natural products occurrence database

			KNApSAcK Species-Metabolite Database
			Zhang *et al.,* 2011
Fingerroot	*Boesenbergia rotunda*	3’, 4’, 5, 7- tetramethoxyflavone	LOTUS - the natural products occurrence database
			Tuchinda *et al.,* 2002
			Jaipetch *et al.,* 1983
Kinokuni Kumquat	*Citrus kinokuni*	3’, 4’, 5, 7- tetramethoxyflavone	LOTUS - the natural products occurrence database
Purple Coral Pea	*Hardenbergia violacea*	3’, 4’, 5, 7- tetramethoxyflavone	Natural Product Activity and Species Source (NPASS)
	Campanula choziatowskyi	3’, 4’, 5, 7- tetramethoxyflavone	Natural Product Activity and Species Source (NPASS)
	Beilschmiedia madang	3’, 4’, 5, 7- tetramethoxyflavone	Natural Product Activity and Species Source (NPASS)
	Pterocaulon serrulatum	3’, 4’, 5, 7- tetramethoxyflavone	Natural Product Activity and Species Source (NPASS)
	Preussia terricola	3’, 4’, 5, 7- tetramethoxyflavone	Natural Product Activity and Species Source (NPASS)
Lavender Sage	*Salvia lavanduloides*	3’, 4’, 5, 7- tetramethoxyflavone	Natural Product Activity and Species Source (NPASS)
	Petunia axillaris subsp. parodii	3’, 4’, 5, 7- tetramethoxyflavone	Natural Product Activity and Species Source (NPASS)
	Heliotropium scabrum	3’, 4’, 5, 7- tetramethoxyflavone	Natural Product Activity and Species Source (NPASS)
	Stephania dielsiana	3’, 4’, 5, 7- tetramethoxyflavone	Natural Product Activity and Species Source (NPASS)
Gentian Sage	*Salvia patens*	3’, 4’, 5, 7- tetramethoxyflavone	Natural Product Activity and Species Source (NPASS)
Chinese Pulsatilla	*Pulsatilla chinensis*	3’, 4’, 5, 7- tetramethoxyflavone	Natural Product Activity and Species Source (NPASS)
Sweetbread Milkcap	*Lactarius mitissimus*	3’, 4’, 5, 7- tetramethoxyflavone	Natural Product Activity and Species Source (NPASS)
European Hop-Hornbeam	*Ostrya carpinifolia (European hop-hornbeam)*	3’, 4’, 5, 7- tetramethoxyflavone	Natural Product Activity and Species Source (NPASS)

Blue Vervain	*Salvia virgata*	3’, 4’, 5, 7- tetramethoxyflavone	Natural Product Activity and Species Source (NPASS)
Northern Pike	*Esox lucius (northern pike)*	3’, 4’, 5, 7- tetramethoxyflavone	Natural Product Activity and Species Source (NPASS)
	Maclura cochinchinensis	3’, 4’, 5, 7- tetramethoxyflavone	Natural Product Activity and Species Source (NPASS)
Douglas Fir Tussock Moth	*Orgyia pseudotsugata (Douglas fir tussock moth)*	3’, 4’, 5, 7- tetramethoxyflavone	Natural Product Activity and Species Source (NPASS)
Blackjack Oak	*Quercus marilandica*	3’, 4’, 5, 7- tetramethoxyflavone	Natural Product Activity and Species Source (NPASS)
Mandarin Orange	*Citrus reticulata*	3’, 4’, 5, 7- tetramethoxyflavone	KNApSAcK Species-Metabolite Database<br>LOTUS - the natural products occurrence database
Ginger	*Zingiber officinale*	3’, 4’, 5, 7- tetramethoxyflavone	KNApSAcK Species-Metabolite Database
	Bryobium eriaeoides	3’, 4’, 5, 7- tetramethoxyflavone	Asamenew *et al.,* 2019<br>LOTUS - the natural products occurrence database
	Dialium excelsum	3’, 4’, 5, 7- tetramethoxyflavone	Natural Product Activity and Species Source (NPASS)
Blazingstar	*Mentzelia decapetala*	3’, 4’, 5, 7- tetramethoxyflavone	Natural Product Activity and Species Source (NPASS)
Urban's Pitcher Sage	*Lepechinia urbanii*	3’, 4’, 5, 7- tetramethoxyflavone	Natural Product Activity and Species Source (NPASS)
	Piper porphyrophyllum	3’, 4’, 5, 7- tetramethoxyflavone	Rajudin *et al.,* 2010.<br>LOTUS - the natural products occurrence database
Rough Starthistle	*Centaurea scoparia*	3’, 4’, 5, 7- tetramethoxyflavone	Natural Product Activity and Species Source (NPASS)
	Fadogia agrestis	3’, 4’, 5, 7- tetramethoxyflavone	Natural Product Activity and Species Source (NPASS)
Calamondin	*Citrus x aurantium f. deliciosa*	3’, 4’, 5, 7- tetramethoxyflavone	LOTUS - the natural products occurrence database
	Parmotrema nilgherrense	3’, 4’, 5, 7- tetramethoxyflavone	Natural Product Activity and Species Source (NPASS)

	Thalassiosira weissflogii	3', 4', 5, 7- tetramethoxyflavone	Natural Product Activity and Species Source (NPASS)
Hound's Tongue	*Cynoglossum macrostylum*	3', 4', 5, 7- tetramethoxyflavone	Natural Product Activity and Species Source (NPASS)
Heartleaf Drymaria	*Drymaria cordata*	3', 4', 5, 7- tetramethoxyflavone	Natural Product Activity and Species Source (NPASS)
Variegated Monkshood	*Aconitum variegatum*	3', 4', 5, 7- tetramethoxyflavone	Natural Product Activity and Species Source (NPASS)
Tangerine	*Citrus tangerina*	3', 4', 5, 7- tetramethoxyflavone	Kim *et al.,* 2009
Orange Jessamine	*Murraya exotica*		Wu *et al.,* 2018

Reference:

1. Fraser, A.W.; Lewis, J.R. Eupatorin, a constituent of *Merrillia caloxylon*. *Planta Med*. **1974**, 1*3(8)*, 1561–1564. doi.org/10.1016/0031-9422(74)80328-6.
2. Azuma, T.; Tanaka, Y.; Kikuzaki, H. Phenolic glycosides from *Kaempferia parviflora*. *Phytochemistry*. **2008**, *69(15),* 2743–2748. doi: 10.1016/j.phytochem.2008.09.001.
3. Chaipech, S.; Morikawa, T.; Ninomiya, K.; Yoshikawa, M.; Pongpiriyadacha, Y.; Hayakawa, T.; Muraoka, O. Structures of two new phenolic glycosides, kaempferiaosides A and B, and hepatoprotective constituents from the rhizomes of *Kaempferia parviflor*a. *Chem Pharm Bull (Tokyo)*. **2012**, *60(1)*, 62–69. doi: 10.1248/cpb.60.62.
4. Azuma, T.; Tanaka, Y.; Kikuzaki, H. Phenolic glycosides from *Kaempferia parviflora*. *Phytochemistry*. **2008**, *69(15)*, 2743-2748. doi: 10.1016/j.phytochem.2008.09.001.
5. Mizuno, M.; Iinuma, M.; Ohara, M.; Tanaka, T.; Iwamasa, M. Chemotaxonomy of the Genus Citrus Based on Polymethoxyflavones. *Chem Pharm Bull (Tokyo)*. **1991**, *39*, 945–949. doi.org/10.1248/cpb.39.945.

6. Zhang, J.Y.; Li, N.; Che, Y.Y.; Zhang, Y.; Liang, S.X.; Zhao, M.B.; Jiang, Y.; Tu, P.F. Characterization of seventy polymethoxylated flavonoids (PMFs) in the leaves of *Murraya paniculata* by on-line high-performance liquid chromatography coupled to photodiode array detection and electrospray tandem mass spectrometry. *J Pharm Biomed Anal*. **2011**, *56(5)*, 950–961. doi: 10.1016/j.jpba.2011.08.019.
7. Tuchinda, P.; Reutrakul, V.; Claeson, P.; Pongprayoon, U.; Sematongz, T.; Santisuk, T.; Taylor, W.C. Anti-inflammatory cyclohexenyl chalcone derivatives in Boesenbergia pandurata. *Phytochemistry*. **2002**, *59(2)*, 169–173. doi: 10.1016/s0031-9422(01)00451-4.
8. Jaipetch, T.; Reutrakul, V.; Tuntiwachwuttikul, P.; Santisuk, T. Flavonoids in the black rhizomes of Boesenbergia panduta. *Phytochemistry*. **1983**. *22(2)*, 625–626. doi.org/10.1016/0031-9422(83)83075-1.
9. Asamenew, G.; Kim, H.W.; Lee, M.K. Characterization of phenolic compounds from normal ginger (Zingiber officinale Rosc.) and black ginger (*Kaempferia parviflora* Wall.) using UPLC–DAD–QToF–MS. *Eur Food Res Technol*. **2019**, *245*, 653–665. doi.org/10.1007/s00217-018-3188-z.
10. Rajudin, E.; Ahmad, F.; Sirat, H.M.; Arbain, D.; Aboul-Enein, H.Y. Chemical constituents from tiger's betel, *Piper porphyrophyllum* N.E.Br. (*Fam. Piperaceae*). *Nat Prod Res*. **2010**, *24(4)*, 387–390. doi: 10.1080/14786410903421826.
11. Kim, Y.D.; Ko, W.J.; Koh, K.S.; Jeon, Y.J.; Kim, S.H. Composition of Flavonoids and Antioxidative Activity from Juice of Jeju Native Citrus Fruits during Maturation. *Korean J Nutr*. **2009**, *42(3)*, 278–290. doi.org/10.4163/kjn.2009.42.3.278.
12. Lu, W.C.; Sheen, J.F.; Hwang, L.S.; Wei, G.J. Identification of 5,7,3',4'-tetramethoxyflavone metabolites in rat urine by the isotope-labeling method and ultrahigh-performance liquid chromatography-electrospray ionization-mass spectrometry. *J Agric Food Chem*. **2012**, *60(33)*, 8123–8128. doi: 10.1021/jf302043a.
13. Fourie, T.G.; du Preez, I.C.; Roux, D.G.; 3',4',7,8-tetrahydroxyflavonoids from the heartwood of *Acacia nigrescens* and their conversion products. *Phytochemistry*. **1972**, *11(5)*, 1763–1770.
14. Li, S.M.; Yang, J.L.; Liu Y.P.; Fu, Y.H. Studies on non-alkaloid constituents from *Alstonia mairei*. *Chinese Tradit Herb Drugs*. **2015**, *24*, 2683–2688.

Table S2. Chemical-Disease Co-Occurrences that can be prevented by 5,7,3',4'-tetramethoxyflavone.

**Decease**	***In vivo/In vitro***	**Form of tetramethoxyflavone studied**	**Mechanism**	**Reference**
inflammatory diseases	*In vitro*: human mast cells (LAD2)	5,7,3',4'-tetramethoxyflavone	Increases gene expression and release of tumor necrosis factor, C-X-C motif chemokine ligand 8, and vascular endothelial growth factor.	Patel *et al.*, 2017
allergic and inflammatory conditions.	*In vitro*: primary human cord blood-derived cultured mast cells	5,7,3',4'-tetramethoxyflavone	Inhibit intracellular calcium level increases and nuclear factor κB induction	Weng *et al.*, 2015
inflammatory diseases	*In vitro*: human mast cells (LAD2) and primary mast cells (hCBMCs)	5,7,3',4'-tetramethoxyflavone	Release of CCL5 and CCL2 and inhibit the activation of p38α MAPK, JNK, and IκB-α	Bawazeer *et al.*, 2019
osteoarthritis	*In vivo*: OA rat models	5,7,3',4'-tetramethoxyflavone	Inhibiting both EP/cAMP/PKA signaling pathway and β-catenin signaling pathway	Wu *et al.*, 2014
osteoarthritis	*In vitro*: C28/I2 cells.	5,7,3',4'-tetramethoxyflavone	Cholesterol dysregulation by increasing the expression of FOXO3a/LXRα/ABCA1 signaling through SIRT1	Peng *et al.*, (2021)

antiinflammatory	*In vivo*: mice	3’, 4’, 7, 8-tetramethoxyflavone	Inhibit synovial human recombinant phospholipase A2 activity, human platelet TXB2 generation, and human neutrophil degranulation.	Ballesteros *et al*., 1995
osteoarthritis	*In vivo*: rats' knee cartilages	3’, 4’, 7, 8-tetramethoxyflavone	activating the expression of IRE1α	Wu *et al*., 2018
inflammatory diseases	*In vitro*: human mast cells (LAD2)	3’, 4’, 7, 8-tetramethoxyflavone	Interaction between neurokinin-1 and ST2 receptors antagonists and siRNA inhibits TNF secretion	Taracanova *et al*., 2017
neurodegenerative diseases	*In vitro*: mast cells and microglia	3’, 4’, 7, 8-tetramethoxyflavone	Inhibit corticotropin-releasing hormone, neurotensin, and substance P	Theoharides *et al*., 2018
allergies, atopic dermatitis, chronic urticaria, cutaneous mastocytosis, and psoriasis	*In vivo*: mastocytosis or mast cell activation syndrome patients	3’, 4’, 7, 8-tetramethoxyflavone	Antiallergic and anti-inflammatory actions	Theoharides *et al*., 2017
Osteoarthritis	*In vivo*: osteoarthritis rat models	3’, 4’, 7, 8-tetramethoxyflavone	Inhibiting both EP/cAMP/PKA signaling pathway and β-catenin signaling pathway	Wu *et al*., 2014
Osteoarthritis	*In vitro*: C28/I2 cells	3’, 4’, 7, 8-tetramethoxyflavone	Increasing the expression of FOXO3a/LXRα/ABCA1 signaling through SIRT1 and ameliorates cholesterol dysregulation	Peng *et al*., 2021
Osteoarthritis	*In vivo*: rat knee osteoarthritis models	3’, 4’, 7, 8-tetramethoxyflavone	Up regulating Foxo3a expression and subsequently inhibiting miR-29a/Wnt/β-catenin signaling activity	Huang *et al*., 2019
Osteoarthritis	*In vitro*: osteoarthritic chondrocytes	3’, 4’, 7, 8-tetramethoxyflavone	Inhibiting PGE2-induced ERS and GSK-3β	Yang *et al*., 2015
Osteoarthritis	*In vitro*: chondrocyte	3’, 4’, 7, 8-	Inhibition of Endoplasmic reticulum stress-induced	Yuan *et al*.,

		tetramethoxyflavone	apoptosis with down regulation of GSK-3β	2017
Psoriasis	*In vitro*: human HaCaT and normal epidermal human keratinocytes	3’, 4’, 7, 8-tetramethoxyflavone	Inhibiting tumor necrosis factor stimulates skin inflammation via mTOR (pmTORSer2448 , pp70S6KThr389 and p4EBP1Thr37/46) signalling.	Patel *et al*., 2018
Anti-inflammatory	*In vitro*: RAW264.7 cells		Inhibited expression of iNOS mRNA and iNOS protein	Sae-Wong *et al*., 2011
Amyotrophic lateral sclerosis	*In vivo*: amyotrophic lateral sclerosis mouse model (TgSOD1 mice)	3’, 4’, 7, 8-tetramethoxyflavone	Reduced degranulation of mast cells in the tibialis anterior muscle	Theoharides *et al*., 2020
Gout	*In vitro*: xanthine oxidase	3’, 4’, 7, 8-tetramethoxyflavone	Inhibitory xanthine oxidase	Nakao *et al*., 2011
Benign prostate hyperplasia	*In vivo*: castrated mice	3’, 4’, 7, 8-tetramethoxyflavone	Suppressed the weights of prostates and seminal vesicles	Murata *et al*., 2013

Reference:

1. Ballesteros, J.F.; Sanz, M.J.; Ubeda, A.; Miranda, M.A.; Iborra, S.; Payá, M.; Alcaraz, M.J. Synthesis and pharmacological evaluation of 2'-hydroxychalcones and flavones as inhibitors of inflammatory mediators generation. *J Med Chem*. **1995**, *38(14)*, 2794–2797. doi: 10.1021/jm00014a032. PMID: 7629818.
2. Peng, F.; Huang, X.; Shi, W.; Xiao, Y.; Jin, Q.; Li, L.; Xu, D.; Wu, L. 5,7,3',4'-tetramethoxyflavone ameliorates cholesterol dysregulation by mediating SIRT1/FOXO3a/ABCA1 signaling in osteoarthritis chondrocytes. *Future Med Chem*. **2021**, *13(24)*, 2153–2166. doi: 10.4155/fmc-2021-0247.
3. Wu, L.; Liu, H.; Li, L.; Liu, H.; Yang, K.; Liu, Z.; Huang, H. 5,7,3',4'-Tetramethoxyflavone exhibits chondroprotective activity by targeting

β-catenin signaling in vivo and in vitro. *Biochem Biophys Res Commun*. **2014**, *452(3)*, 682–688. doi: 10.1016/j.bbrc.2014.08.129.
4. Wu, L.; Liu, H.; Li, L.; Xu, D.; Gao, Y.; Guan, Y.; Chen, Q. 5,7,3',4'-Tetramethoxyflavone protects chondrocytes from ER stress-induced apoptosis through regulation of the IRE1α pathway. *Connect Tissue Res*. **2018**,*59(2)*, 157–166. doi: 10.1080/03008207.2017.1321639.
5. Weng, Z.; Patel, A.B.; Panagiotidou, S.; Theoharides, T.C. The novel flavone tetramethoxyluteolin is a potent inhibitor of human mast cells. *J Allergy Clin Immunol*. **2015**, *135(4)*, 1044–1052.e5. doi: 10.1016/j.jaci.2014.10.032.
6. Bawazeer, M.A.; Theoharides, T.C. IL-33 stimulates human mast cell release of CCL5 and CCL2 via MAPK and NF-κB, inhibited by methoxyluteolin. *Eur J Pharmacol*, **2019**, *865*,172760. doi: 10.1016/j.ejphar.2019.172760.
7. Patel, A.B.; Theoharides, T.C. Methoxyluteolin Inhibits Neuropeptide-stimulated Proinflammatory Mediator Release via mTOR Activation from Human Mast Cells. *J Pharmacol Exp Ther*. **2017**, *361(3)*, 462–471. doi: 10.1124/jpet.117.240564.
8. Taracanova, A.; Alevizos, M.; Karagkouni, A.; Weng, Z.; Norwitz, E.; Conti, P.; Leeman, S.E.; Theoharides, T.C. SP and IL-33 together markedly enhance TNF synthesis and secretion from human mast cells mediated by the interaction of their receptors. *Proc Natl Acad Sci USA*. **2017**, *114(20)*, E4002–E4009. doi: 10.1073/pnas.1524845114.
9. Theoharides, T.C.; Tsilioni, I. Tetramethoxyluteolin for the Treatment of Neurodegenerative Diseases. *Curr Top Med Chem*. **2018**, *18(21)*, 1872–1882. doi: 10.2174/1568026617666181119154247.
10. Theoharides, T.C.; Stewart, J.M.; Tsilioni, I. Tolerability and benefit of a tetramethoxyluteolin-containing skin lotion. *Int J Immunopathol Ph*. **2017**, *30(2)*, 146–151. doi: 10.1177/0394632017707610.
11. Wu, L.; Liu, H.; Li, L.; Liu, H.; Yang, K.; Liu, Z.; Huang, H. 5,7,3',4'-Tetramethoxyflavone exhibits chondroprotective activity by targeting β-catenin signaling in vivo and in vitro. *Biochem Biophys Res Commun*. **2014**, *452(3)*, 682–688. doi: 10.1016/j.bbrc.2014.08.129.
12. Peng, F.; Huang, X.; Shi, W.; Xiao, Y.; Jin, Q.; Li, L.; Xu, D.; Wu, L. 5,7,3',4'-tetramethoxyflavone ameliorates cholesterol dysregulation by mediating SIRT1/FOXO3a/ABCA1 signaling in osteoarthritis chondrocytes. *Future Med Chem*. **2021**, *13(24)*, 2153–2166. doi: 10.4155/fmc-2021-0247.
13. Huang, X.; Chen, Z.; Shi, W.; Zhang, R.; Li, L.; Liu, H.; Wu, L. TMF inhibits miR-29a/Wnt/β-catenin signaling through upregulating Foxo3a activity in osteoarthritis chondrocytes. *Drug Des Devel Ther*. **2019**, *19(13)*, 2009–2019. doi: 10.2147/DDDT.S209694.

14. Yang, J.; Liu, H.; Li, L.; Liu, H.; Shi, W, Wu, L. The Chondroprotective Role of TMF in PGE2-Induced Apoptosis Associating with Endoplasmic Reticulum Stress. *Evid-Based Complement Altern Med*. **2015**. *2015*, 297423. doi: 10.1155/2015/297423.
15. Yuan, X.; Li, L.; Shi, W.; Liu, H.; Huang, X.; Liu, Z.; Wu, L. TMF protects chondrocytes from ER stress-induced apoptosis by down-regulating GSK-3β. *Biomed Pharmacother*. **2017**, *89*, 1262–1268. doi: 10.1016/j.biopha.2017.03.028.
16. Patel, A.B.; Tsilioni, I.; Weng, Z.; Theoharides, T.C. TNF stimulates IL-6, CXCL8 and VEGF secretion from human keratinocytes via activation of mTOR, inhibited by tetramethoxyluteolin. *Exp Dermatol*. **2018**, *27(2)*, 135–143. doi: 10.1111/exd.13461.
17. Sae-Wong, C.; Matsuda, H.; Tewtrakul, S.; Tansakul, P.; Nakamura, S.; Nomura, Y.; Yoshikawa, M. Suppressive effects of methoxyflavonoids isolated from *Kaempferia parviflora* on inducible nitric oxide synthase (iNOS) expression in RAW 264.7 cells. *J Ethnopharmacol*. **2011**, *136(3)*, 488–495. doi: 10.1016/j.jep.2011.01.013.
18. Theoharides, T.C.; Tsilioni, I. Amyotrophic Lateral Sclerosis, Neuroinflammation, and Cromolyn. *Clin Ther*. **2020**, *42(3)*, 546–549. doi: 10.1016/j.clinthera.2020.01.010.
19. Nakao, K.; Murata, K.; Deguchiz, T.; Itoh, K.; Fujita, T.; Higashino, M.; Yoshioka, Y.; Matsumura, S.; Tanaka, R.; Shinada, T.; Ohfune, Y.; Matsuda, H. Xanthine oxidase inhibitory activities and crystal structures of methoxyflavones from *Kaempferia parviflora* rhizome. *Biol Pharm Bull*. **2011**, *34(7)*, 1143–1146. doi: 10.1248/bpb.34.1143.
20. Murata, K.; Hayashi, H.; Matsumura, S.; Matsuda, H. Suppression of benign prostate hyperplasia by *Kaempferia parviflora* rhizome. *Pharmacognosy Res*. **2013**, *5(4)*, 309–314. doi: 10.4103/0974-8490.118827.
